# Supplementary material for: CRISPR‐TAPE: protein‐centric CRISPR guide design for targeted proteome engineering
Source: Mol Syst Biol. 2020 Jun 2;16(6):e9475. doi: 10.15252/msb.20209475 (PMC7266498; doi:10.15252/msb.20209475)
Supplement: Supplementary file 1 — Appendix [file MSB-16-e9475-s001.pdf]

Title: CRISPR-TAPE: protein-centric CRISPR guide design for targeted proteome engineering

Authors: Anderson, DP.* [1], Benns, HJ.* [1,2], Tate, EW.[2], Child, MA[1].

* These authors contributed equally: Daniel P Anderson and Henry J Benns

Appendix

- Appendix note

Appendix Note: Use of CRISPR-TAPE (application-associated README document)

Overview

CRISPR-TAPE is a python-based CRISPR design tool for TArgeted Protein Engineering, available as a standalone application for Windows and macOS operating systems. The CRISPR-TAPE source code is open source and their modularity allows for easy incorporation of additional functions (github.com/LaboratoryChild/CRISPR-TAPE). Users may achieve enhanced CPU times using the raw python scripts and this is recommended for computationally intensive guide RNA (gRNA) generation (e.g. for genomes larger than 1 giga-base). The standard CRISPR-TAPE application incorporates a custom graphic user interface (GUI) and is recommended for most users working with genomes less than one giga-base in size.

Motivation

Existing CRIPSR gRNA design tools non-specifically target the entire input region of DNA. Current gene-centric design algorithms fail to consider protein-focused users. CRISPR-TAPE has been developed to reduce the substantial time burden associated with manual curation of gRNA libraries, and make CRISPR-based protein modification strategies more accessible to the protein engineering community.

Current version

CRISPR-TAPE version 1.0.0

System requirements

Systems running CRISPR-TAPE must meet the following requirements:

- macOS version 10.9.5 or later, 4 GB+ RAM
- Windows 7 or later, 4 GB+ RAM

For target organisms with genomes greater than one giga-base in size, it is recommended systems have at least 32 GB RAM to prevent application crashes, with 64 GB+ RAM recommended for genomes greater than three giga-bases in size (e.g. *Homo sapiens*).

Installation

The following provides and overview for the installation process. A detailed walk-through with example sequences is provided in the "**Walk-through and test example sequences**" section below.

- CRISPR-TAPE for Windows and macOS is available from via http://www.laboratorychild.com/crispr-tape

⇒ For Windows-based systems, download and unzip the CRISPR-TAPE.exe file and place it in a directory of your choice.

⇒ On macOS systems, the CRISPR-TAPE.dmg should be downloaded, mounted, and the CRISPR-TAPE application file dragged and dropped into the Application alias folder when prompted.

- For successful gRNA identification, the genome of the organism of interest must be located within a single .txt file titled "INSERT_ORGANISM_GENOME_HERE.txt". Genome files are typically available for download in FASTA format, and can be directly renamed "INSERT_ORGANISM_GENOME_HERE.txt". This renamed file can then be moved to the appropriate file directory (detailed above), replacing the exiting file in that location.

⇒ On Windows, this file must be located within the same directory as the CRISPR-TAPE.exe file.

⇒ On macOS the file is located within the CRISPR-TAPE.app package via the following path: "./CRISPR-TAPE.app/Contents/Resources", accessible by right clicking the application and pressing "Show Package Contents" => subdirectory "Contents" => subdirectory "Resources".

These respective Windows and macOS directories are also where gRNA .csv files are outputted to once the programme has run successfully.

Input

Within the CRISPR-TAPE GUI, users input the filename of the outputted .csv file and the genomic loci and coding sequences of the protein of interest. Users then specify the protospacer adjacent motif (PAM) sequence via a checkbox. To target a specific residue, users input the numerical position of the residue within the protein sequence and the maximum distance for gRNA identification if desired, then choose "Run Option 1". To target all amino acids of one type, input the single letter code of the residue of interest and choose "Run Option 2".

Output

The list of the gRNAs generated by CRISPR-TAPE are outputted to a ".csv" file specified by the user within the CRISPR-TAPE home directory on Windows and within "./CRISPR-TAPE.app/Contents/Resources" on macOS.

The dataframe outputted by Specific_function consists of:

1) gRNA Sequence: The gRNA sequence identified by the programme.
2) PAM: The specific protospacer adjacent motif immediately adjacent to the gRNA
3) Strand: The orientation of the DNA strand the gRNA targets relative to the sense of the inputted genomic loci.

4) G/C Content: The percentage of the gRNA sequence consisting of “G” and “C” bases.
5) Distance from aa (bp):
   - If positive and “Strand” = forward: gRNA is upstream of the amino acid, the distance is measured from the base on the right-hand side of the nuclease cut site to the base on the left-hand side of the codon (5´to 3´).
   - If negative and “Strand” = forward: gRNA is downstream of the amino acid, the distance is measured from the base on the left-hand side of the nuclease cut site to the base on the right-hand side of the codon (5´to 3´).
   - If positive and “Strand” = reverse: gRNA is upstream of the amino acid, the distance is measured from the base on the left-hand side of the nuclease cut site to the base on the left-hand side of the codon (5´to 3´)
   - If negative and “Strand” = reverse strand: gRNA is downstream of the amino acid, the distance is measured from the base on the right-hand side of the nuclease cut site to the base on the right-hand side of the codon (5´to 3´).
6) Notes: Does the gRNA contain a poly-T sequence indicated by a tandem of 4 or more Ts? Does the gRNA have a leading G at position 1 in the gRNA? Is the G/C content over 75%?
7) Off-target Count: The number of off-target sites the gRNA may target.

The dataframe outputted by General_function consists of:
1) Amino Acid Position: The position of the amino acid within the amino acid sequence.
2) Adjacent amino acids: The 4 amino acids immediately surrounding the residue being targeted. The target residue is indicated by “*”
3) 5´ gRNA Sequence: The sequence of the gRNA closest in proximity upstream of the amino acid.
4) 3´ gRNA Sequence: The sequence of the gRNA closest in proximity downstream of the amino acid.
5) PAM: The specific protospacer adjacent motif immediately adjacent to the 5´ or 3´ gRNA.
6) Strand: The orientation of the DNA strand the 5´ or 3´ gRNA targets relative to the sense of the inputted genomic loci.
7) G/C Content: The percentage of the 5´ or 3´ gRNA sequence consisting of “G” and “C” bases.
8) Distance of cut site from Amino Acid (bp):
   - If “Strand” = forward and the gRNA is 5´ of the residue: The distance is measured from the base on the right-hand side of the nuclease cut site to the base on the left-hand side of the codon (5´to 3´).
   - If “Strand” = forward and the gRNA is 3´ of the residue: The distance is measured from the base on the left-hand side of the nuclease cut site to the base on the right-hand side of the codon (5´to 3´).

- If “Strand” = reverse and the gRNA is 5´ of the residue: The distance is measured from the base on the left-hand side of the nuclease cut site to the base on the left-hand side of the codon (5´to 3´).
- If “Strand” = reverse and the gRNA is 3´ of the residue: The distance is measured from the base on the right-hand side of the nuclease cut site to the base on the right-hand side of the codon (5´to 3´).

9) Notes: Does the 5´ or 3´ gRNA contain a poly-T sequence indicated by a tandem of 4 or more Ts? Does the gRNA have a leading G at position 1 in the gRNA? Is the G/C content over 75%?
10) Off-target Count: The number of off-target sites the 5´ and 3´ gRNA may target.

Walk-through and test example sequences

Here, we provide a basic walk-though for new users, including test input sequences for the *Toxoplasma gondii* gene TGGT1_242330. The outputs from user tests can be directly compared against those in Figure 2 in the associated manuscript (see **Citation** below).

Step:

1. CRISPR-TAPE comes preloaded with the *Toxoplasma gondii* GT1 genome (release 46). The genome file was downloaded in FASTA format and directly renamed “INSERT_ORGANISM_GENOME_HERE.txt”. This file was then moved to the appropriate file directory (detailed in **Installation** above), replacing the existing file in that location.

2. Following installation launch the CRISPR-TAPE application.

3. Choose a file name for the gRNA outputs, and type in the input box: “**Name of guide output file (no spaces)**”. For this example, name the file “**test**”.

4. Copy-paste the following sequence into the “**Please input the genomic loci sequence of your protein here**” box. Note that the gene intron-exon structure is distinguished though the use of lower- and uppercase, respectively. The gene sequence used for this test example does not include UTRs:

ATGGCGACCGAGGCGAAGCTTTTCGGCCGCTGGTCGTACGACGATGTCAACGTCAGCGACC
TTTCGCTCGTTGACTACATCGCGGTCAAGGACAAGGCCTGCGTCTTCGTGCCGCACACTGCT
GGACGCTACCAGAAGAAGAGATTCCGCAAGGCCATGTGCCCCATTGTCGAGCGTCTCGTCAA
CTCCATGATGATGCACGGCCGgtatgttgtgcgtccgaagataagcggttgataggagctacttgcctttctccggagactctt
gaaccctcaagaatccgatccgtgtgcgcggccgagcaactgtgtgctccatggggagagagcggaacacgcgtattcatgtatctgtgt
attataatgcatgcgtctgtcgccggtcatcgcgcgaggcgccgagatgttagtgagggggaacatgggagtgccctctggccccggata
gagtagtttcttgttgagtagggatccgttgtgtttcatgtttttttacagCAACAACGGGAAGAAAACTCTCTCTGTGCGCA

TCGTCCGCCACGCCTTCGAAATCATCCACCTGATGACGGACAAGAACCCCATTCAGGTCTTC
GTCAACGCTGTTGAGAATGGCGGCCCCCGTGAAGACAGCACTCGAATCGGATCTGCTGGTGT
TGTCAGACGCCAGGCTGTTGATGTGTCTCCCCTCCGCAGAGTGAACCAGGCCATCTACCTCA
TCTGCACCGGCGCAAGgtcagtccctctgaaagaaactgtacagctcctcacagagcggcagcgcgtggggtgttgaggtgt
gtgtcagttcgtttgctgcacagtcagctgttgagtagtcgtcgctcttcaagatccgtccggcgtttgcgagtctctgccccagtctttcgtgaa
gcgtagagtcaacggcatgggtggatgtttttcagGCTGGCGGCCTTCCGGAACATCAAGACCATCGCCGAGT
GCCTTGCGGACGAGATCATGAACTGCGCCAAGGAGTCCTCCAACGCTTACGCGATAAAGAAG
AAGGACGAAATCGAGCGTGTTGCGAAGGCAAACCGATAA

5. UTRs are not necessary to successfully identify gRNAs, and so the 5´ and 3´ UTR input boxes can be left empty for this example.

6. Copy-paste the following sequence into the "**Please input the coding sequence of your protein here (no UTRs)**" box:

ATGGCGACCGAGGCGAAGCTTTTCGGCCGCTGGTCGTACGACGATGTCAACGTCAGCGACC
TTTCGCTCGTTGACTACATCGCGGTCAAGGACAAGGCCTGCGTCTTCGTGCCGCACACTGCT
GGACGCTACCAGAAGAAGAGATTCCGCAAGGCCATGTGCCCCATTGTCGAGCGTCTCGTCAA
CTCCATGATGATGCACGGCCGCAACAACGGGAAGAAAACTCTCTCTGTGCGCATCGTCCGCC
ACGCCTTCGAAATCATCCACCTGATGACGGACAAGAACCCCATTCAGGTCTTCGTCAACGCT
GTTGAGAATGGCGGCCCCCGTGAAGACAGCACTCGAATCGGATCTGCTGGTGTTGTCAGAC
GCCAGGCTGTTGATGTGTCTCCCCTCCGCAGAGTGAACCAGGCCATCTACCTCATCTGCACC
GGCGCAAGGCTGGCGGCCTTCCGGAACATCAAGACCATCGCCGAGTGCCTTGCGGACGAGA
TCATGAACTGCGCCAAGGAGTCCTCCAACGCTTACGCGATAAAGAAGAAGGACGAAATCGAG
CGTGTTGCGAAGGCAAACCGATAA

7. Select "**NGG**" when asked to "**Specify nuclease protospacer adjacent motif (PAM)**".

8. Initially testing **OPTION 1**: In the "**Input the residue position of this amino acid**" box type "**143**".

9. In the "**Please specify the maximum guide distance (in nucleotides) from the amino acid**" box type "**30**".

10. Then click the "**Run Option 1**" box. A pop-up box will appear informing you when "**Your guide RNAs have successfully been generated**". For Windows-based systems, the output .csv file is located within the same directory as the CRISPR-TAPE.exe file. On macOS the output .csv file is located within the CRISPR-TAPE.app package itself. This is accessible by right-clicking

on the application and selecting “Show Package Contents” => subdirectory “Contents” => subdirectory “Resources”. The output .csv file from this test of Option 1 will be named “test” (see step 3 of this walk-through). The output file should match that shown in Figure 2a of the associated manuscript (see **Citation** below).

11. To test **OPTION 2**, amend the file name (see step 3 above) to “**test02**”. If the filename is not changed, the new output file overwrites the old output file. There is no need to change the inputs currently present in any of the other boxes as each run mode (Option 1 or 2) executes independently.

12. In the “**Input your target amino acid single letter code**” box, type “**C**”. Then click “**Run Option 2**”. This will identify the closest suitable gRNA 5´ and 3´ of each cysteine within the input coding sequence.

13. The successful identification of gRNA will be announced, and can be located as described in step 10 above. The output file should match that shown in Figure 2b of the associated manuscript (see **Citation** below).

Troubleshooting

- All inputs are case sensitive, and it is important to adhere to the requirements specified adjacent to each entry box.
- gRNAs will be incorrect if exonic bases are not capitalised and intronic/untranslated bases are not lowercase in the genomic loci input.
- The programme currently only recognises standard “A”, “T”, “C” and “G” base nomenclature, and will filter out any other characters.
- If no protospacer adjacent motif is specified, the programme will not run and no guide RNAs will be outputted.
- The programme will stop running and a pop-up prompt will open if the inputted protein coding sequence and the exon sequences from the inputted genomic loci do not match.
- Off target counts will display as “-1” if gRNAs are not found within the forward or reverse complement of the organism genome. If this occurs, ensure the genomic loci and genome originate from the same organism.
- If some amino acids of a given type are missing from the OPTION 2 output, this is because they cannot be targeted using the current information. If this is the case, add more upstream and downstream bases to the input requesting 100 bases upstream and downstream of the genomic loci.

- Crashes are likely the result of your system not meeting the minimum system requirements (see **System requirements** above). In our experience this only occurs when working with genome greater than one giga-base in size.
- It is possible that in certain unanticipated user situations error-pops might not appear, indicating a new error that has not been previously identified. Please report as requested for bugs (see below).

Error/bug reporting

We encourage all users to report any bugs they discover via the contact form at http://www.laboratorychild.com/contact
