# Supplementary material for: CRISPR‐TAPE: protein‐centric CRISPR guide design for targeted proteome engineering
Source: Mol Syst Biol. 2020 Jun 2;16(6):e9475. doi: 10.15252/msb.20209475 (PMC7266498; doi:10.15252/msb.20209475)
Supplement: Supplementary file 2 — Expanded View Figures PDF [file MSB-16-e9475-s002.pdf]

Expanded View Figures

Figure EV1. Graphic user interface (GUI) of CRISPR-TAPE.

Standalone application interface of CRISPR-TAPE allowing users to query gRNAs targeting specific amino acid residues or amino acid types.

 © 2020 The Authors

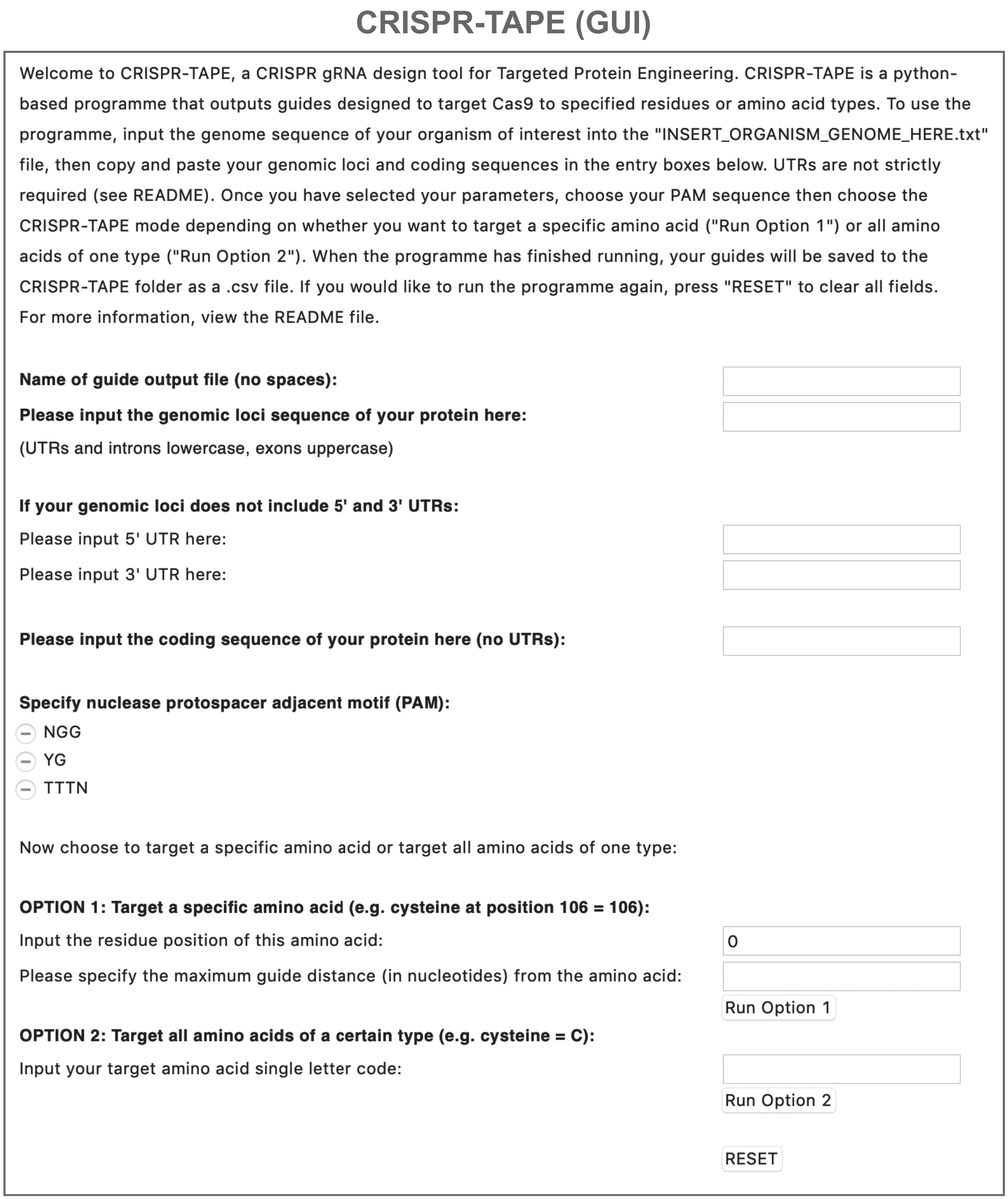
CRISPR-TAPE (GUI)

Welcome to CRISPR-TAPE, a CRISPR gRNA design tool for Targeted Protein Engineering. CRISPR-TAPE is a python-based programme that outputs guides designed to target Cas9 to specified residues or amino acid types. To use the programme, input the genome sequence of your organism of interest into the "INSERT_ORGANISM_GENOME_HERE.txt" file, then copy and paste your genomic loci and coding sequences in the entry boxes below. UTRs are not strictly required (see README). Once you have selected your parameters, choose your PAM sequence then choose the CRISPR-TAPE mode depending on whether you want to target a specific amino acid ("Run Option 1") or all amino acids of one type ("Run Option 2"). When the programme has finished running, your guides will be saved to the CRISPR-TAPE folder as a .csv file. If you would like to run the programme again, press "RESET" to clear all fields. For more information, view the README file.

Name of guide output file (no spaces):

Please input the genomic loci sequence of your protein here:
(UTRs and introns lowercase, exons uppercase)

If your genomic loci does not include 5' and 3' UTRs:
Please input 5' UTR here:
Please input 3' UTR here:

Please input the coding sequence of your protein here (no UTRs):

Specify nuclease protospacer adjacent motif (PAM):
NGG
YG
TTTN

Now choose to target a specific amino acid or target all amino acids of one type:

OPTION 1: Target a specific amino acid (e.g. cysteine at position 106 = 106):
Input the residue position of this amino acid: 0
Please specify the maximum guide distance (in nucleotides) from the amino acid:
Run Option 1

OPTION 2: Target all amino acids of a certain type (e.g. cysteine = C):
Input your target amino acid single letter code:
Run Option 2

RESET

Figure EV1.

© 2020 The Authors

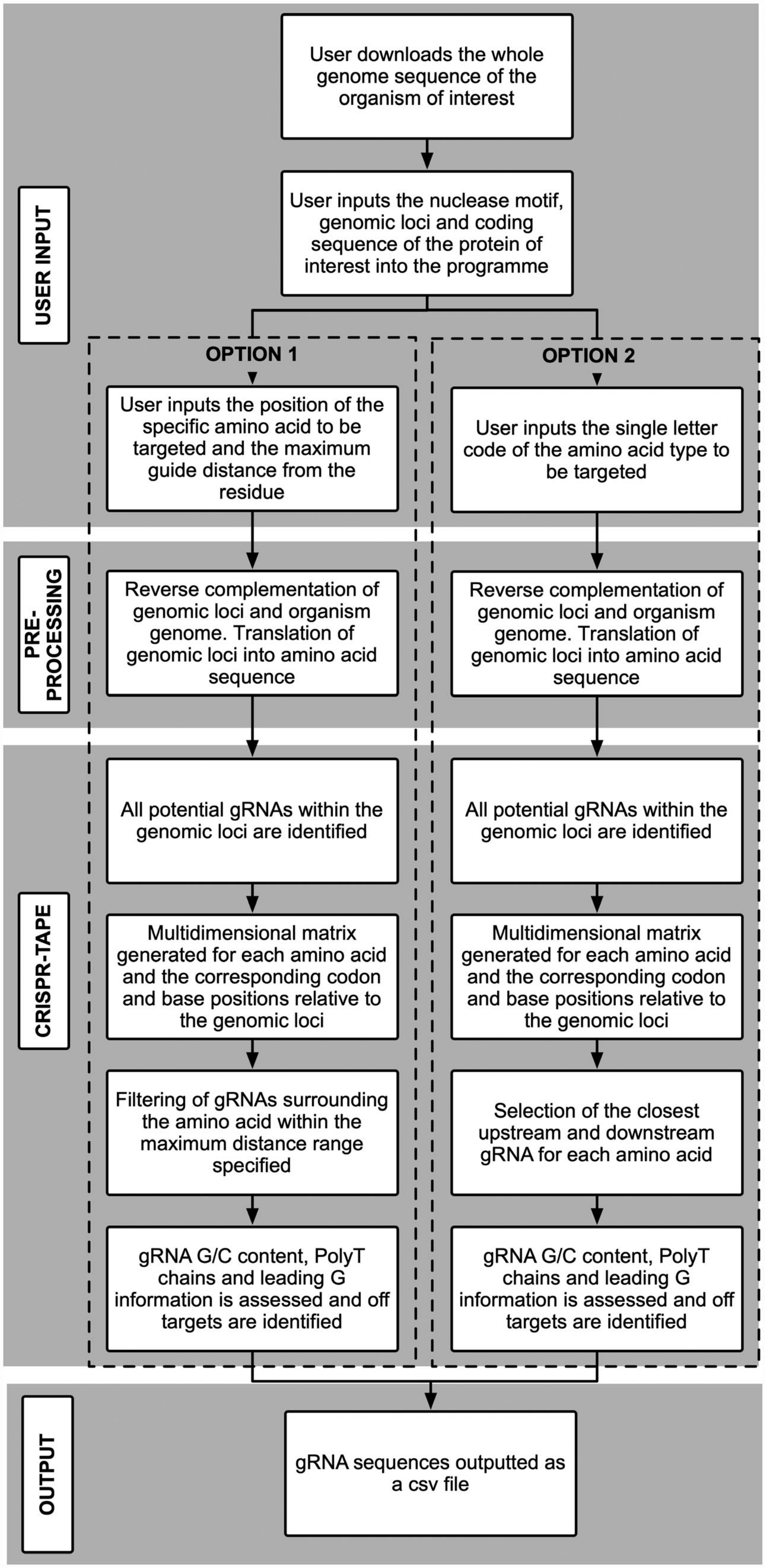

Figure EV2. Flowchart schematic of CRISPR-TAPE algorithm.

CRISPR-TAPE algorithm depicted as simple box workflow broken down into: user inputs, pre-processing steps, CRISPR-TAPE matrix mapping, and outputs. Workflow provided for both position-specific and type-specific queries (OPTION 1 and 2 respectively).

© 2020 The Authors

Figure EV3. CRISPR-TAPE performance comparisons.

A Left: comparison of processing speeds of CRISPR-TAPE versus total gRNA output for genes from the latest *Toxoplasma gondii* GT1 genome release (ToxoDB-46). Mean (± SD) gRNA output processing times are displayed for three genes of various CDS lengths (n = 3) with “NGG” specified as the target protospacer adjacent motif (PAM). For the position-specific function, gRNAs were queried within a distance of 30 nucleotides (nt) from methionine at position 1. For the type-specific function, leucine was specified as the target amino acid. Right: minimum, maximum and mean processing rates for each function across the same dataset.

B Linear regression analyses of amino acid frequency and CDS length across the gene panel analysed in A. Coefficients of determination (R^2) are indicated for each amino acid type.

© 2020 The Authors

A

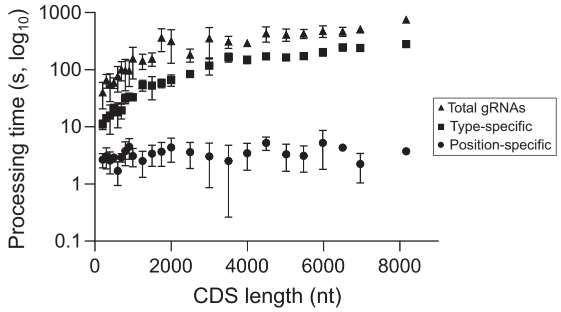

Processing rate (nt s^{-1})	Total gRNAs	Type-specific	Position-specific
Min:	3.22	15.52	58.45
Max:	22.69	71.39	7902.73
Average:	8.43	26.76	330.76

B

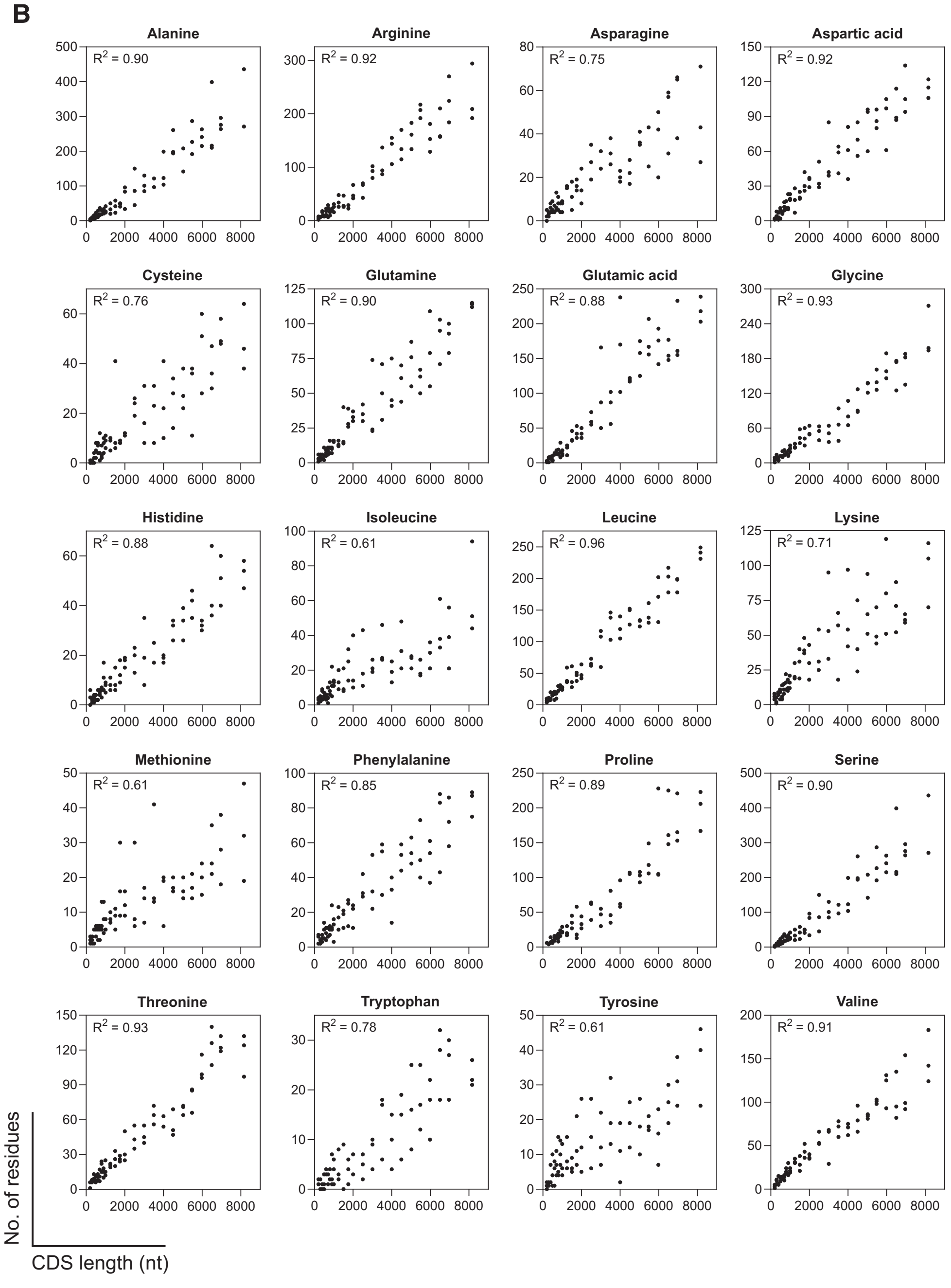

Figure EV3.

 © 2020 The Authors
