# Supplementary material for: CRISPR‐TAPE: protein‐centric CRISPR guide design for targeted proteome engineering
Source: Mol Syst Biol. 2020 Jun 2;16(6):e9475. doi: 10.15252/msb.20209475 (PMC7266498; doi:10.15252/msb.20209475)

**Table EV2:** Application of CRISPR-TAPE to generate gRNAs targeting palmitoylated cysteines in *Plasmodium falciparum.*


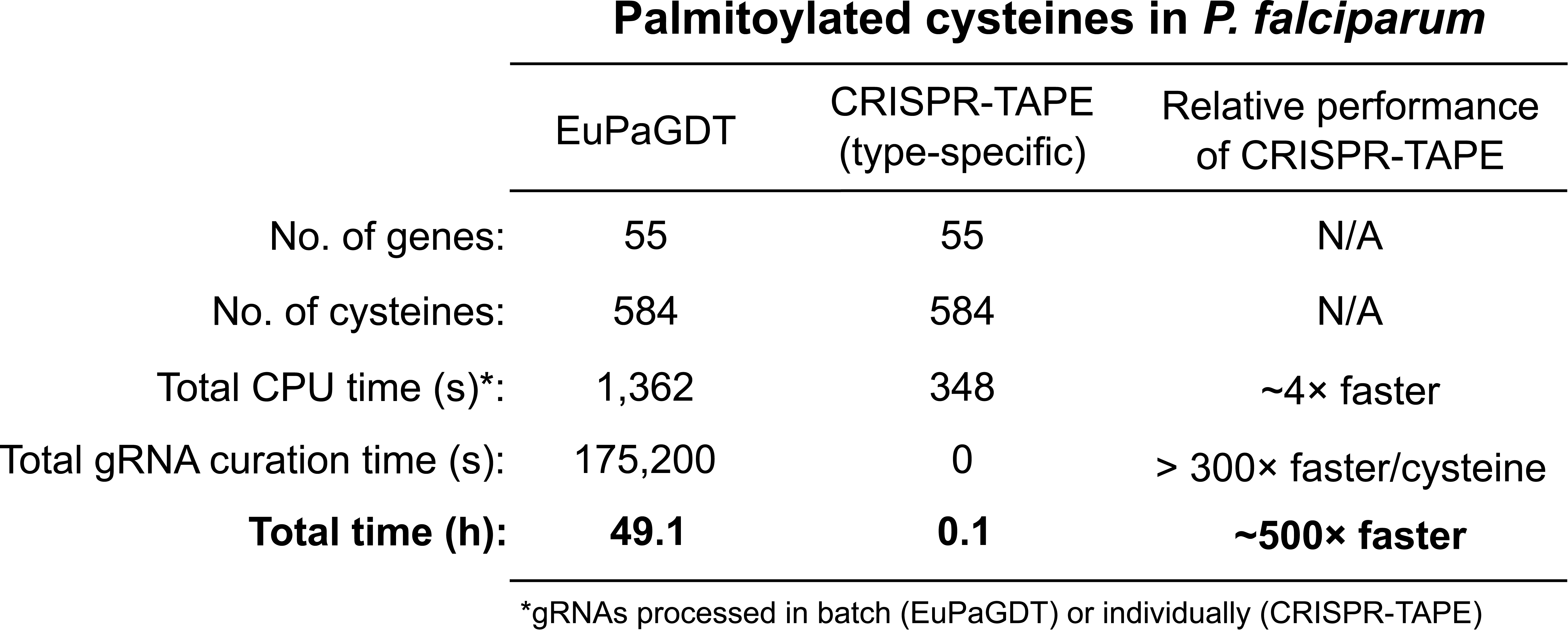

Supplement: Supplementary file 4 — Table EV2 [file MSB-16-e9475-s004.docx]
