# Supplementary material for: CRISPR‐TAPE: protein‐centric CRISPR guide design for targeted proteome engineering
Source: Mol Syst Biol. 2020 Jun 2;16(6):e9475. doi: 10.15252/msb.20209475 (PMC7266498; doi:10.15252/msb.20209475)

**Table EV3:** Application of CRISPR-TAPE to generate gRNAs targeting ligandable kinase-associated reactive lysines in *Homo sapiens*.


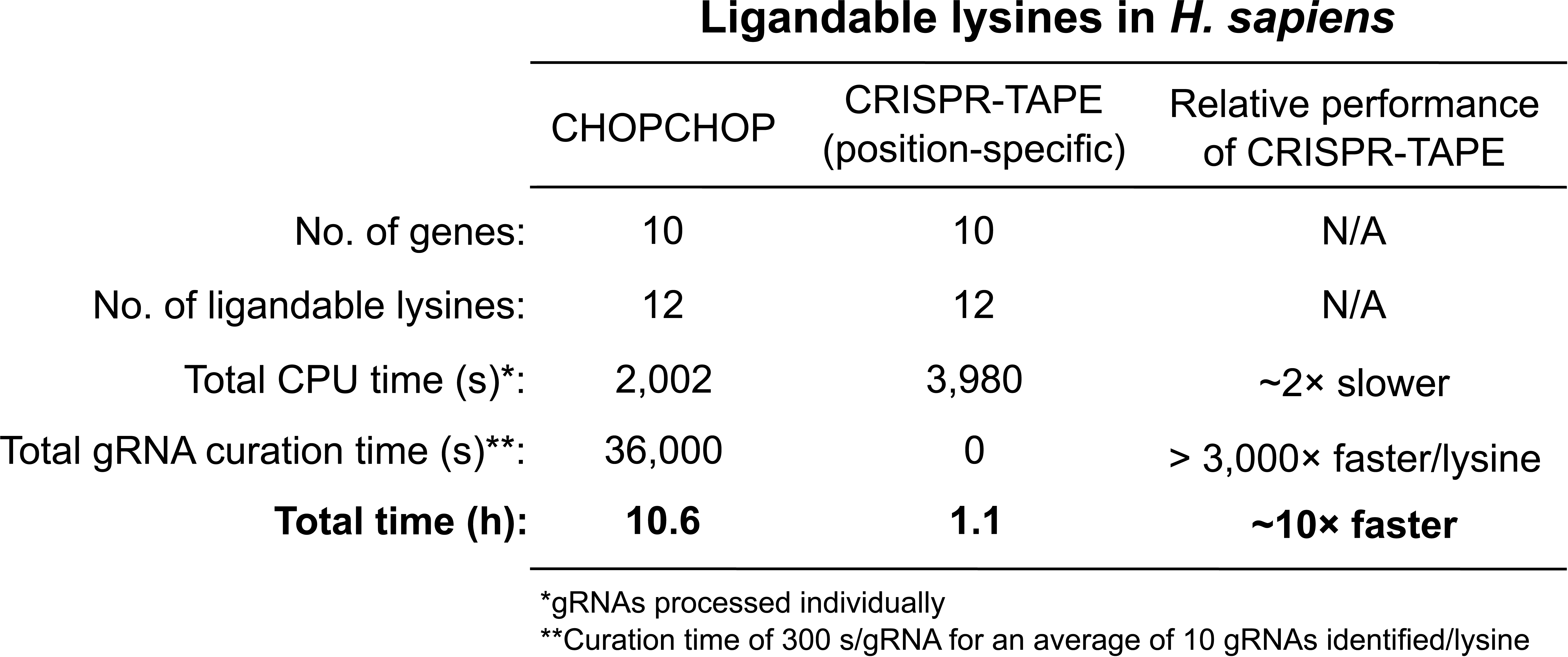

Supplement: Supplementary file 5 — Table EV3 [file MSB-16-e9475-s005.docx]
